# Traumatic Diabetes

**Published:** 1859-11

**Authors:** 


*Traumatic Diabetes.*—Dr. Plagge reports a case brought on in a young man by a blow on the occiput, which, besides a swelling, seemed to produce no effect. In two or three days, however, the youth was seized with amblyopia, thirst, and craving for food, while he passed a large quantity of urine which yielded much sugar. There was no uneasiness about the livers nor jaundice. Tannin and opium together, with flesh diet, were prescribed during a week, with little effect, three or four quarts of urine being passed daily. Under the use of bicarb. of soda, with flesh diet, however, the sugar gradually disappeared from the urine, although this fluid continued to be passed in too great abundance, as simple polyuria, for two month, longer.—*Med. Times and Gaz.*, June 11, 1859, from *Virchow Archiv.* B. xiii.

